# Non-target digital ischemia in an ulnar artery distribution following transradial access: Case report and review of best practice techniques

**DOI:** 10.1177/11297298211000897

**Published:** 2021-03-14

**Authors:** Patrick Kennedy, Darren Klass, John Chung

**Affiliations:** 1 Department of Medical Imaging, Faculty of Medicine, University of Toronto, Toronto, ON, Canada; 2 Department of Medical Imaging, North York General Hospital, Toronto, ON, Canada; 3 Department of Radiology, Faculty of Medicine, University of British Columbia, Vancouver, BC, Canada; 4 Division of Interventional Radiology, Department of Radiology, Vancouver General Hospital, Vancouver, BC, Canada

**Keywords:** Interventional radiology, techniques and procedures, transradial, vascular access, thromboembolism, digital ischemia, complication

## Abstract

Transradial access is a safe approach for visceral endovascular interventions, with lower complication rates compared to transfemoral access. This report describes an unusual case of ulnar artery thrombosis following splenic artery aneurysm embolization via left transradial approach, resulting in non-target digital ischemia and eventual amputation of the ring and little finger distal phalanges. Technical considerations to reduce the incidence of access complications are also reviewed, along with practice modifications undertaken at our institution following this case to improve outcomes.

## Introduction

Greater adoption of transradial access (TRA) in interventional radiology (IR) is based on robust literature describing lower mortality, bleeding, and access complications in TRA versus transfemoral access (TFA).^1–3^ There is a substantial patient preference for TRA, with one study reporting that 98% of patients who had experienced both TFA and TRA interventions would choose TRA for subsequent procedures.^4^ In morbidly obese patients, TRA is considered safe and feasible, carrying a lower hemostatic device failure rate compared to TFA.^5^

The largest randomized trial comparing the two approaches describes a rate of major complications related to TRA of 0.4%.^2^ Possible complications include symptomatic vasospasm, pseudoaneurysm formation, dissection, hematoma without underlying vascular injury, radial artery occlusion (RAO), and digital ischemia.^2–7^ While deaths have occurred from bleeding related to TFA, there have been no known cases of death in the literature related to TRA site complications.

Performing TRA procedures requires adequate training, equipment, and infrastructure to prevent and manage complications. To reduce the risk of RAO, tumescent anesthesia should be administered prior to gaining access, followed by administration of intra-arterial antispasmodic medication once access is achieved. Rates of RAO can also be reduced with prophylactic ulnar compression upon completion of the procedure to promote radial artery patency.^8^ The main reason to prevent RAO is not to decrease the hand ischemia risk but to preserve future access.

In this report, we describe a case of ulnar artery thrombosis and digital ischemia following splenic artery aneurysm embolization via left TRA with eventual need for digital amputation, a complication not previously described in the literature. The research ethics board at our institution waives formal approval for case reports. The individual described in this report provided informed consent for publication of patient information and images.

## Case description

A 47-year-old woman presented for elective embolization of a splenic artery aneurysm. The patient is a Jehovah’s Witness with underlying obesity. Possible risks of the procedure were discussed, including access site complications, and informed consent was obtained. A bedside Barbeau test was performed on the left wrist, demonstrating a type A wave form, and TRA was therefore offered. The left arm was prepped and draped and ultrasound-guided left TRA was obtained. Access was secured with a five French low-profile hydrophilic sheath. An antispasmodic cocktail (nitroglycerin 200 mcg, verapamil 2.5 mg, and heparin 2000 units) was instilled through the sidearm of the sheath, which was then closed. Embolization was performed through a five French base catheter and 2.8 French microcatheter using detachable coils and ethylene vinyl alcohol liquid embolic. Patent hemostasis was achieved upon sheath removal.

The patient described left forearm and hand pain immediately post-procedure. On examination, the radial and ulnar pulses were palpable. Doppler signals were present in the palmar arch and digits and bedside ultrasound confirmed that the radial artery was patent (the ulnar artery was not assessed with ultrasound). Given these findings, radial artery spasm was suspected. The patient was monitored overnight and discharged the following morning with improvement in pain.

**Figure 1.**
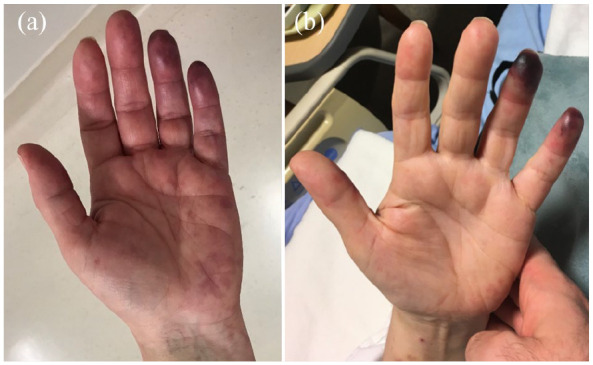
Photographs of the patient’s left hand demonstrating the evolution of digital ischemia, with (a) cyanotic patches on the distal index and little fingers as well as the hypothenar eminence 3 days post-procedure and (b) progression to necrosis of the tips of the index and little fingers 12 days post-procedure.

**Figure 2.**
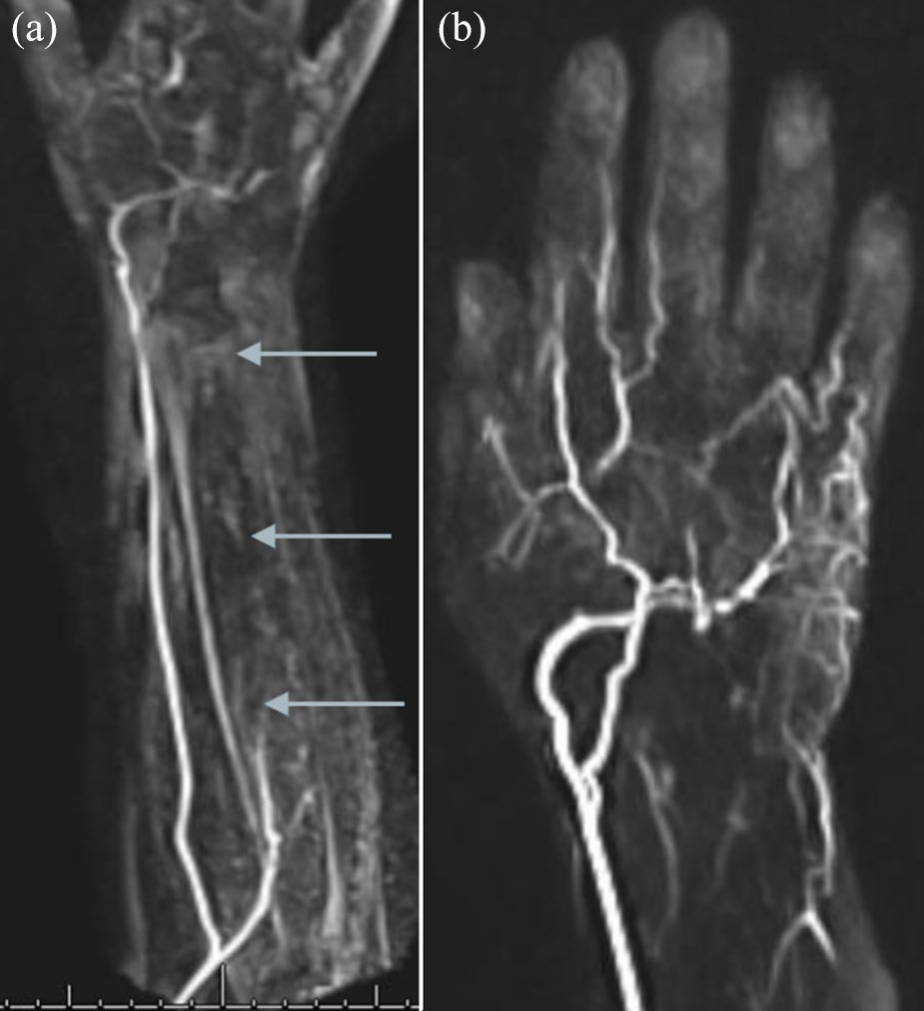
Time-resolved magnetic resonance angiogram of the forearm (a) and hand (b) obtained 4 days post-procedure demonstrating an absence of contrast filling of the ulnar artery beyond its proximal segment (arrows), the palmar arches supplied by the radial artery, and poor contrast filling of the fourth and fifth digital arteries.

On day three post-procedure, she presented with worsening left hand pain and numbness in an ulnar distribution. Cyanosis of the distal ring and little fingers was observed (Figure 1(a)). Computed tomography (CT) angiography demonstrated good contrast filling of the brachial and radial arteries but not the ulnar artery. Management was initiated including a heparin infusion, warm compresses, transdermal nitroglycerin paste, and analgesia. A time-resolved magnetic resonance angiogram was performed on day four, demonstrating no flow in the ulnar artery beyond its proximal segment (Figure 2(a)). The palmar arches were supplied by the radial artery, with poor flow into the ring and little finger digital arteries (Figure 2(b)).

**Figure 3.**
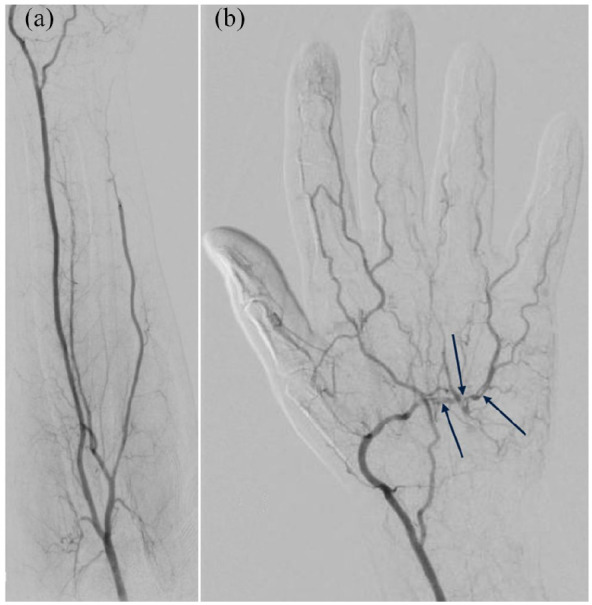
Catheter angiography of the forearm (a) and hand (b) performed on post-procedure day 12 demonstrating improved patency of the ulnar artery in its proximal and mid segments but no flow in its distal segment, nonocclusive filling defects in the deep palmar arch suggestive of thromboembolism (arrows), and ongoing poor contrast filling of the fourth and fifth digital arteries.

The imaging findings and clinical presentation were reviewed by IR and vascular surgery and a decision was made to avoid catheter-directed thrombolysis due to the risk of distal embolism and potential worsening of ischemia. On day seven, CT-guided sympathetic nerve root block was performed to treat potential vasospasm contributing to the ulnar artery occlusion, without improvement in cyanosis or pain. Thoracoscopic sympathectomy was then undertaken 2 days later, with temporary improvement in pain but no improvement in cyanosis. Catheter angiography was performed on post-procedure day 12 via TFA. This demonstrated ongoing distal ulnar artery occlusion and a patent radial artery (Figure 3(a)). On left hand angiography, poor contrast filling of the ring and little finger digital arteries as well as small defects in the deep palmar arch due to thromboembolism were observed (Figure 3(b)).

Given the ongoing digital ischemia, brachial-to-ulnar bypass grafting was performed. Unfortunately, there was no clinical improvement, with evolution to necrosis of the fourth and fifth fingertips (Figure 1b). Plastic surgery was consulted for wound management and consideration of amputation. Surgical amputation of the distal portions of the ring and little fingers was performed one-month post-embolization. Apart from expected psychological and emotional challenges, there were no other clinical concerns on follow-up one-month post-amputation.

## Conclusions

Despite the excellent safety profile and general patient preference for TRA over TFA for visceral endovascular interventions, the procedure is not without risk. TRA was selected for this patient given its lower complication rate, which continues to be the approach and philosophy at our institution. Specific to this patient’s case, her obesity inferred a higher risk of life-threatening hemorrhage from TFA while her Jehovah’s Witness status (which precludes blood transfusions) would have compounded the situation in the event of any major TFA-related hemorrhage.

This case outlines a major complication with a devastating outcome for the patient. Although transient digital ischemia has been previously described,^6^ there are no reports of digital necrosis requiring amputation. Further, associated ulnar artery thrombosis in the context of a widely patent radial artery is not described in the literature neither clinically nor mechanistically. In the context of conventional forearm arterial anatomy and standard TRA sheath and catheter position in this case, the cause for ulnar artery thrombosis and distal thromboembolism is not clear. One possible mechanism is the presence of an elongated thrombus on the tip of the sheath, dislodged upon sheath removal and following the path of least resistance into the ulnar artery during radial artery compression. Other possibilities include distal embolism of a thrombus on the tip of the sheath upon removal with subsequent proximal extension of thrombus into the ulnar artery, or dislodgement of a thrombus at the tip of the microcatheter or base catheter during removal with embolization into the ulnar artery. Regardless of the true mechanism, this case highlights the importance of proper technique as well as catheter and sheath hygiene to minimize the risk of complications associated with vascular occlusion and thromboembolism. In addition, the digital ischemia was caused by emboli, as demonstrated on the arm and hand angiogram performed following the complication, rather than an isolated RAO which is significantly more common than distal embolization. It is imperative to ensure best practice is followed after any TRA procedure. Adequate doses of heparin should be administered, the catheter should be flushed and preferably removed over a wire, and the sheath must be aspirated following removal of the catheter to ensure there is no thrombus at the tip or in the sheath itself. Otherwise, application of the compression band will cause the sheath to collapse and any unaspirated thrombus could potentially embolize into the forearm or hand circulation. If the sheath is flushed without aspirating first, thrombus could be injected into the forearm circulation.

Of note, continuous sheath flushing was abandoned as routine practice at our center in 2016 due to lack of data showing improved sheath patency, provided there is adequate sheath and catheter hygiene with aspiration and flushing prior to removal. A continuous sheath flush with heparinized saline is established if there is a mismatch between sheath and catheter French size. Continuous flushing is also established for catheters and microcatheters if the risk of clinically significant distal embolization at the target site is high (e.g. in pulmonary arteriovenous malformation embolization).

The post-procedure management of this patient was undertaken in a collaborative, multidisciplinary manner. Even so, strategies for further optimizing patient care and clinical outcomes in IR can be taken from this case. Although catheter-directed pharmacomechanical thrombolysis was not performed to avoid this risk of distal embolism and worsening of digital ischemia, this treatment may have slowed or even reversed the development of ischemia and necrosis. Similarly, earlier surgical intervention with brachial-to-ulnar bypass grafting may have improved flow to the digital arteries supplied by the ulnar artery thereby avoiding the need for amputation. As the first description of TRA-related ulnar artery thrombosis and digital ischemia requiring amputation, the described case highlights the importance of early diagnosis and potentially earlier, more aggressive management strategies to reduce the risk of access site complications with long term sequelae. Many highly experienced operators around the world were asked for management advice, but no consensus was reached on any single strategy to manage this complication, highlighting the rarity of this complication.

Following this case, a review was undertaken of all women age 18–60 years who underwent TRA for visceral and peripheral endovascular interventions at our institution over 5 years. A total of 225 procedures were identified. Nine (4.0%) access site complications occurred, with eight (3.6%) minor complications not requiring invasive treatment and not resulting in long term sequelae. The case described in this report was identified as the one major complication in the review, yielding an institution-specific major complication rate of 0.4%.

In an effort to reduce the incidence of clinically significant thrombosis following TRA procedures, we have also introduced practice modifications at our institution as a result of this case. Specifically, the heparin dose in the intra-arterial antispasmodic cocktail has been altered from 2000 units to weight-based dosing at 50 units/kg for procedures using four or five French sheaths, and 75 units/kg for procedures using six French or larger sheaths. We have standardized the heparin dose for uterine artery embolization to 75 units/kg due to the inherently higher rate of radial spasm in younger woman and therefore the probable higher rate of RAO in this specific population. We have also standardized the administration of an additional 2000 units of heparin every 60 min of intra-arterial procedure time.

In conclusion, digital ischemia is a rare complication from TRA; however, it may have devastating consequences for patients. Adequate heparin dosing and sheath hygiene are essential to reduce the risk of non-target digital ischemia.
